# Volatile-Mediated Attraction of Greenhouse Whitefly *Trialeurodes vaporariorum* to Tomato and Eggplant

**DOI:** 10.3389/fpls.2017.01285

**Published:** 2017-07-20

**Authors:** Hewa L. C. Darshanee, Hui Ren, Nazeer Ahmed, Zhan-Feng Zhang, Yan-Hong Liu, Tong-Xian Liu

**Affiliations:** ^1^State Key Laboratory of Crop Stress Biology for Arid Areas, Northwest A&F University Yangling, China; ^2^Key Laboratory of Integrated Pest Management on the Loess Plateau of Ministry of Agriculture, Northwest A&F University Yangling, China; ^3^Department of Export Agriculture Kandy, Sri Lanka

**Keywords:** *Trialeurodes vaporariorum*, tomato plant, eggplant, conspecific-infested plants, headspace volatiles, SPME

## Abstract

The behavior of the greenhouse whitefly, *Trialeurodes vaporariorum* Westwood (Hemiptera: Aleyrodidae), is known to be affected by plant volatile cues, but its attraction or repellent to specific volatile cues has not been deeply studied yet. Therefore, the aim of our study was to identify the most attractive plant among cultivars of tomato (*Solanum lycopersicum*) and eggplant (*Solanum melongena*) to evaluate the volatiles of plants to identify the chemical compound(s) that attract *T. vaporariorum*. We speculated that whitefly–host plant interaction primarily depends on plant volatile emissions and that once the plant is damaged, it might attract more whiteflies. Three intact (uninfested) tomato, four intact eggplant cultivars and whitefly infested plants of the most whitefly attractive tomato and eggplant cultivars were examined by behavioral assay experiments for attractiveness to *T. vaporariorum* and headspace volatile were determined by solid-phase microextraction (SPME) and gas chromatography-mass spectrometry. Whiteflies had the highest preference for the intact eggplant Kuai Yuan Qie (KYQ) among the eggplant and the tomato plant cultivars in bioassay experiments. Although both male and female whiteflies were significantly more attracted to infested KYQ plants than to intact plants, whitefly females did not select conspecific-infested YG plants. The volatile emissions among different plant cultivars in individual species and infested versus intact plants were significantly different. Among these volatiles, identified major green leaf volatiles [(Z)-3-hexen-1-ol] and terpenoids [α-pinene, (E)-β-caryophyllene, α-humulene, azulene] showed a constitutive relationship with the most whitefly preference plants. Our findings provide new insights into the chemical compounds that attract or repel whiteflies.

## Introduction

In agro-ecosystems, plants and herbivores interact with plant metabolites either directly or indirectly. Volatile-mediated foraging behavior is prominent in herbivore pests when they target host plants (Dicke and Baldwin, 2010). In natural conditions, plants produce different blends of volatiles like monoterpenes, sesquiterpenes, aldehydes, and green leaf volatiles (GLVs) (Rajabaskar et al., 2013), whose chemical composition can strongly depend on plant varieties or cultivars within an individual species (McDaniel et al., 2016) as well as the plant’s age (Quintero and Bowers, 2011). In addition, plants release large quantities of volatile organic compounds (VOCs) known as herbivore-induced plant volatiles (HIPVs) after infestation by herbivores, which provide foraging cues for the same or different types of herbivores and their natural enemies (Dicke and Baldwin, 2010; Xiao et al., 2012). Many studies have shown that the behavioral responses of herbivores to their host plants largely depend on volatile-mediated interactions from a distance, even without the visual stimuli of the plants (Bleeker et al., 2009, 2011).

Performance and preference of whiteflies (Hemiptera: Aleyrodidae) on host plants are remarkably different among plant species owing to the diversity of plant morphological, physiological, and chemical characteristics. Eggplant (*Solanum melongena* L.) attracts significantly higher numbers of the whitefly *Trialeurodes vaporariorum* Westwood than do pepper and poinsettia plants (Lee et al., 2010; Moreau and Isman, 2010). Additionally, the potential abundance of *T. vaporariorum* is higher on tomato (*Solanum lycopersicum* L.) than on pepper (*Capsicum annuum* L.) (Lorenzo et al., 2016). However, even for the same plant species, whitefly preference is not the same across different cultivars. *Trialeurodes vaporariorum* is highly attracted to some commercially available tomato cultivars as compared with wild tomato species (*Solanum habrochaites* and *Lycopersicon pimpinellifolium* L.) (Lucatti et al., 2010; McDaniel et al., 2016). This variable attraction may depend on the VOC properties of the eggplant and tomato cultivars, as the chemical compounds manipulating the behavioral responses of *T. vaporariorum* are less known except their attraction studied for the sexual pheromone (Yin and Maschwitz, 1983) and tomato leaf volatiles (Tsueda et al., 2014).

A semiochemical-based push–pull approach, using VOCs as both attractant and repellent, might be one strategy to reduce whitefly abundance in field conditions. *Trialeurodes vaporariorum* has been one of the most destructive whitefly species because their feeding behavior directly or indirectly causes yield losses in many kinds of field crops, greenhouse crops, and ornamental plants worldwide (Lee et al., 2010; Moreau and Isman, 2010). However, recent control strategies for *T. vaporariorum* are grounded in different approaches, including chemical, biological, cultural, and mechanical techniques (Moreau and Isman, 2010; Hernandez et al., 2013; Bresch et al., 2014). Synthetic chemical pesticides have been widely used to reduce the harmful effects of *T. vaporariorum* on crop growth and final yield, though unfortunately control of this pest remains a major challenge due to their resistance development, rapid reproduction, and behavior including settling beneath the leaf surface (Erdogan et al., 2012; Ovcarenko et al., 2014). Furthermore, most attention is given to their negative effects on natural enemies (Sohrabi et al., 2013) and the environment (Norin, 2007).

A novel approach to global pest management is the usage of semiochemicals in crop protection while reducing chemical pesticide application, because semiochemicals almost certainly have minimal impact on non-target and beneficial insects and natural ecosystems. Use of semiochemicals to reduce pest damage varies according to their capacity to change the behavior of target insects. We hypothesized that whitefly attraction primarily depends on plant volatile emission and we therefore attempted to determine the most attractive plant cultivars for *T. vaporariorum* among the most commonly grown eggplant and tomato cultivars. SOS signals are produced by herbivore-damaged plants to attract natural enemies of the herbivores (Dicke and Baldwin, 2010; Gols et al., 2015). A few studies have shown that plants exploit chemical signals that provide foraging cues for conspecific or heterospecific herbivores after herbivore feeding (Ameline et al., 2007; Zhang et al., 2009; Dicke and Baldwin, 2010). Based on this premise, we speculated that the volatiles from conspecific-infested eggplant and tomato plants might alter the aggregation behavior of *T. vaporariorum* compared to that at intact plants and analysis of chemical compounds from the most whitefly attractive intact plant and infested plant may lead to identification of the attractive volatile compounds.

## Materials and Methods

### Insect Rearing

The greenhouse whitefly, *Trialeurodes vaporariorum*, that was previously identified was collected from insectaries in the Key Laboratory of Applied Entomology, Northwest A&F University, Yangling, Shaanxi, China and reared on tobacco [*Nicotiana tabacum* L. (Solanaceae) var. Qinyan 95] plants in screen cages (60 cm × 60 cm × 60 cm) at 25 ± 2°C, 65 ± 5% relative humidity (RH) and a photoperiod of 16L:8D. The newly emerged *T. vaporariorum* adults were transferred to a new screen cage. The procedure was practiced up to four generations and then, adults were used in all experiments without considering their ages. To sex separation, whiteflies were directly exposed to carbon dioxide gas for 10 s to anesthetize them. Males and females were separated using a tiny paint brush under the binocular microscope based on the shape of their abdomen end and body size. They were then transferred to two separated petri dishes (1.5 cm height × 8 cm diameter) and kept for 30 min for ensuring they are active during the Y-tube olfactometer bioassays.

### Plant Materials

Tomato *Solanum lycopersicum* ‘Chuan Zhu Ying Tao’ (CZY) (Hebei Jin Shu Lv Seed Industry Co., Ltd., Shijiazhuang, China), ‘Song Tian Hongmandi’ (STH) (Shaanxi Song Tian Biological Technology Co., Ltd., Xi’an, China), and ‘Yang Guang 906’ (YG) (Shaanxi Sunshine Seed Industry Co., Ltd., Xi’an, China) and eggplant *Solanum melongena* ‘Zi Guan Qie’ (ZGQ) (Shaanxi Qin Xing seed and seedling Co., Ltd., Xi’an, China), and ‘Kuai Yuan Qie’ (KYQ) (Tianjin Geng Yun Seed Industry Co., Ltd., Tianjin, China) were selected because they are the most commonly grown cultivars in China and purchased from a local vegetable seed market at Yangling, Shaanxi, China. However, the new eggplant lines H149 and 899 provided by the Institute of Vegetables, Chongqing Academy of Agricultural Science (Chongqing, China) were also used in our experiment. Some of these tomato and eggplant cultivars have been used in our previous studies as well (Shah and Liu, 2013). The plants were grown in pots (7 cm × 7 cm × 8 cm) in the greenhouse at 25 ± 2°C, 65 ± 5% RH, and a photoperiod of 16L:8D h until 5 weeks from seed sowing. At this stage, heights of the plants were approximately 18 cm and have 4–5 well-developed leaves.

### Infested Plants

To obtain infested plants, 200 adult whiteflies of mixed ages and sexes were collected from the insectaries and introduced into net cages (45 cm × 45 cm × 45 cm) containing an individual plant and kept for 24 h. In the Y-tube olfactometer experiments, infested plants were used with whiteflies, but for the wind tunnel experiments, the whitefly adults and eggs were removed from the plants using a tiny painting brush just before the start of the experiments.

### Intact Plants

A set of 5-week-old plants were kept in clean cages (45 cm × 45 cm × 45 cm) and carefully handled without either herbivore pests or mechanical damage under the same environmental conditions as for the infested plants.

### Two-Choice Bioassays

#### (a) Y-tube Olfactometer Experiments

Bioassays were conducted in a closed system with a two-armed Y-tube olfactometer with approximately 60° between the two arms. Each section of the Y-tube was 80 mm in length with an internal diameter of 8 mm. The experiments were performed between 14:00 and 20:00 h in a dark room at 25 ± 2°C, 65 ± 5% RH. During the bioassays, a 20 W fluorescent light was fixed vertically 0.5 m over the Y-tube olfactometer with maintaining the 65-lux light intensity at the surface of Y-tube olfactometer. Prior to each experiment, all glassware was washed with disinfectant liquid [84 Disinfectant, Sodium Hypochlorite (active chlorine content: 5.5–6.5%), Xian Hongchang Washing Co., Ltd., China] and rinsed with 70% ethyl alcohol, then further washed with distilled water and dried in an oven at 120°C for 2 h. The olfactometer set-up was arranged as described by Koschier et al. (2000) and De Kogel and Van Loon (2003). Purified air was passed through an activated charcoal filter and then humidified with distilled water. The Y-tube was connected to an air pump through flow meters set at 200 mL/min airflow and maintained for 30 min before the start of bioassays. Prior to the experiment, plant pots were covered with 25 cm × 38 cm oven bags (EasyOven polythene bags, Reynolds Kitchens, Lake Forest, IL, United States), which were tied with Teflon tape. In this set-up the above ground part of the plant was only exposed to the air stream that passed through the 3 L glass jars (**Supplementary Figure S1**). In the first bioassay, different plant cultivars within an individual plant species were tested. Then, the bioassay was performed for the most attractive tomato cultivar versus eggplant cultivar. In the third experiment, infested versus intact plants of the most whitefly attractive cultivars within two plant species were examined. *Trialeurodes vaporariorum* were individually released within the first centimeter of the base of Y-tube and observed for 5 min. A choice was made when the insect passed halfway or further into one arm of the Y-tube. If they did not select either arm within the given time, the assay was concluded as no response. After every 10 insects, the Y-tube was washed as previously described and the entire setup of Y-tube with connected jars was rotated to avoid a positional effect. Twenty insects from each sex were tested in every experiment, and all experiments were repeated on five consecutive days at the same time. The entire setup was cleaned before the next set of insect assays was performed; new plants and whiteflies were used each day.

To observe the male and female whitefly response to the chemical components released by their conspecifics, the following Y-tube olfactometer arrangement was made. Two hundred *T. vaporariorum* adults were collected and kept in a 250 mL glass bottle and then connected to the olfactometer as an odor-loaded air against a clean bottle connected as a control. The preference behavior of 20 males and 20 females were individually observed and the experiment was repeated on five consecutive days with new sets of whiteflies.

#### (b) Wind Tunnel Experiment

The bioassay was conducted in a large horizontal glass chamber. Height, length, and width of the wind tunnel were 60, 200, and 60 cm, respectively, with 200 mL/min wind speed, 25 ± 2°C temperature, and 65 ± 5% RH. Plants were placed in the wind tunnel flight chamber for 30 min before release of the whiteflies. The distance from the plants to the whitefly release platform was 100 cm, and the two treatments were arranged opposite each other at the upwind end of the chamber. The release platform level was adjusted according to the plant canopy height. All experiments were repeated five times. After an experiment, the wind tunnel was cleaned with 70% alcohol and kept fully empty for at least 1 h prior to the next experiment. Repetitions used new sets of plants and whiteflies. The two treatments in the wind tunnel were rotated after each test. The plant treatments were used as Y-tube olfactometer experiments. In each experiment, approximately 100 whiteflies were released. Upwind orientation was recorded after 24 h.

### Free-Choice Bioassays

Free-choice experiments with *T. vaporariorum* were performed in net cages (45 cm × 120 cm × 120 cm) at 25 ± 2°C temperature, 65 ± 5% RH and a photoperiod of 16L:8D h. In a single experiment, all plant cultivars of individual species were tested and two plants from each cultivar were taken for set up arrangement. In this set up, at a time, six plants from tomato (Three cultivars × 2) or eight plants from eggplants (Four cultivars × 2) were tested. Plants were randomly arranged in a circle in the cage at similar distances from each other. Whiteflies were released at the center of the circle from a releasing platform; platform height was adjusted according to the plants’ canopy height. For every experiment, 100 whiteflies (mixed sexes) were released at once and the number of whiteflies that had settled on each plant was recorded after 24, 48, and 72 h intervals to evaluate their preference changes across time. All the plants with whiteflies were removed after 72 h and the cage remained fully empty for 24 h prior to arrange next set of plants. A set of experiments for single plant species was repeated at least five times, and the positions for plant cultivars were changed in every repetition to avoid a positional effect.

### Multi-Choice Bioassays

The multi-choice bioassays were conducted in a small greenhouse (800 cm × 500 cm) using three plants from each cultivar per treatment at 25 ± 2°C temperature, 65 ± 5% RH and a photoperiod of 16L:8D h. Four-week-old plants of the two most attractive tomato cultivars and four most attractive eggplant cultivars, as determined by the two-choice and free-choice bioassays, were planted in soil with the potting mixture to prevent damage to the roots. Plants were arranged in the middle of the greenhouse in 100 cm distance from each other in a complete randomized design. After 1 week, 2,000 whiteflies were released from a petri dish which was hung in the center of greenhouse at 100 cm above the ground level. Three days later, the number of whiteflies on each plant was carefully counted. All the plants were removed with whiteflies at the end of experiment. The greenhouse was clean and kept empty for 3 days. The experiment was repeated three times.

### Headspace Volatile Collection and Analysis

Dynamic headspace volatile collection was carried out for intact tomato and eggplant plant volatiles using a solid-phase microextraction (SPME) fiber coated with poly dimethylsiloxane-divinylbenzene (PDMS-DVB, 65 μm; Supelco, Bellefonte, PA, United States). Volatile collection glass jars were washed using washing liquid [84 Disinfectant, Sodium Hypochlorite (active chlorine content: 5.5–6.5%), Xian Hongchang washing Co., Ltd., China], pure ethyl alcohol, and distilled water in succession, and kept in an oven at 200°C for 4 h. Potted plants were arranged with the same method that was used in the olfactometer assay. Then, a single plant was placed in a 3-L glass jar with two openings, one at the bottom and one at the top. These openings were covered with clean aluminum foil, and a small hole was made in the upper-opening foil, using a needle, for the SPME fiber probe. Environmental conditions were set at 25 ± 2°C, 65 ± 5% RH. Prior to the experiment, the SPME fiber was conditioned at 250°C for 30 min in a gas chromatograph (GC) injection port according to the guideline of the manufacturer. After 30 min, an SPME fiber was inserted into the headspace above the plant inside the glass jar to absorb dynamic headspace volatiles. Then, the aluminum foil at the bottom opening was removed, and a Teflon tube affixed to allow purified air, which was filtered via charcoal and then Tenax TA absorbent (80–100 mesh; Scientific Instrument Services, Inc., Ringoes, NJ, United States), to pass through the glass jar; this was maintained for 60 min. In each experiment, plants were used only once and then discarded. To obtain the volatiles from infested plants, first volatiles from undamaged plants were collected as a control. Then the same plant was kept in a net cage with 200 whiteflies (mixed sexes) and maintained at 25 ± 2°C, 65 ± 5% RH and a photoperiod of 16L:8D h for 24 h. The volatiles from infested versus intact plants were collected only for the two most highly attractive cultivars (KYQ and YG).

After 60 min, the SPME needle was carefully taken out and immediately inserted into a gas chromatography-mass spectrometry (GC-MS) thermal desorption port, where the fiber was extended and kept for 5 min. The GC-MS system contained of a GC (TRACE 1310, Thermo Fisher Scientific, Waltham, MA, United States) that was used for the separation of volatile chemicals and an MS (ISQ Single Quadrupole MS, Thermo Fisher Scientific) used for detection, identification, and quantification of volatiles. The thermally desorbed VOCs were separated in a 30 m long, 0.32 mm i.d. and 0.25 μm film thickness HP-5MS UI capillary column (Agilent Technologies, Santa Clara, CA, United States). GC-MS running time was 25 min. The GC was operated in splitless mode with purified helium at a flow of 1.0 mL/min as a carrier gas. The initial GC oven temperature was set to 40°C for 4 min, ramped at 8°C min^-1^ to 250°C, and held for 5 min. MS transfer line and ion source temperature was maintained at 280°C and quadrupole temperature was at 250°C, with a scan range from 33 to 500 amu. The MS was operated in electron ionization (EI) mode. The ion energy and emission current were maintained at 70 eV and 25 μA, respectively. The Xcalibur program (Ver. 2.1, Thermo Electron Corporation, San Jose, CA, United States) was utilized for data acquisition. The identification of separated compounds was carried out by comparing to the NIST 2011 (National Institute of Standards and Technology, Washington, DC, United States) mass spectral library. The peak area of individual components of the volatiles was used to measure the relative quantity.

### Data Analysis

In the two-choice assays, preferences of *T. vaporariorum* in the Y-tube and wind tunnel were analyzed with Chi-square (χ^2^) tests and transformed to percentages. Statistical analysis for free-choice bioassays in the net cages and multi-choice bioassays in the greenhouse were performed by the one-way analysis of variance (ANOVA), where means were separated by the least significant difference test (LSD) (*P* < 0.05). To analyze the volatiles, the peak area of each replicate was proportionate to the quantity of total peak areas of individual component. Individual volatile compounds of three tomato cultivars, four eggplant cultivars, and infested versus intact plants were compared with one-way ANOVAs. Principal component analysis (PCA) was used to determine whether plant cultivars in individual species belonged to separate groups on the basis of relative abundance percentages of volatile compounds. These analyses were performed based on the full data set of 26 major volatile compounds of tomato plants and 21 compounds of eggplants. To observe the differences of volatile emissions from infested and intact plants, further PCA analysis was performed using major seven volatiles of eggplant KYQ and tomato YG. Plants that produced in small amounts of volatile were excluded from analysis. The results were visualized in score plots. IBM SPSS Statistics version 19 (Chicago, IL, United States) was used for all statistical analysis.

## Results

### Two-Choice Assay for Intact Tomato

**FIGURE 1:**
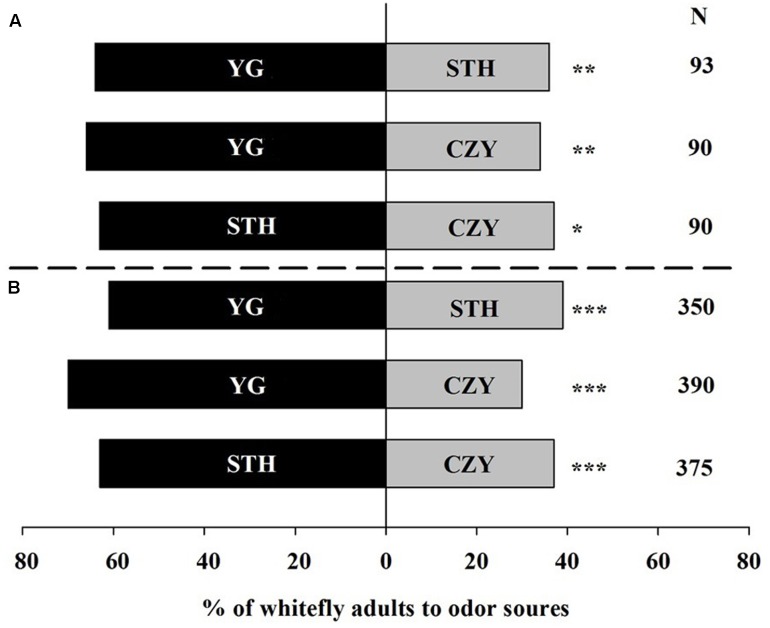
Preference of *Trialeurodes vaporariorum* to three different tomato cultivars in **(A)** Y-tube olfactometer and **(B)** wind tunnel. Odor sources are intact *Solanum lycopersicum* cultivar Chuan Zhu Ying Tao (CZY), Song Tian Hongmandi (STH), and Yang Guang 906 (YG). The numbers to the right of the bars (N) give the number of responding individuals. The asterisks indicate a significant preference for one of the two odor sources (χ^2^ test: ^∗^*P* < 0.05, ^∗∗^*P* < 0.01, ^∗∗∗^*P* < 0.001).

In the Y-tube olfactometer, 91% of the whiteflies (without considering their sexes) responded toward odor sources while 74% whiteflies made a choice toward odors in the wind tunnel bioassays. Overall, the tomato plant cultivar most attractive to whiteflies was YG, followed by STH and CZY, in that order, in the olfactometer (**Figure 1A**). When whiteflies had a choice between YG and STH, 64% of the whiteflies selected YG (χ^2^ = 7.84, *P* = 0.005). In addition, 66% of the whiteflies preferred YG over CZY (χ^2^ = 8.71, *P* = 0.003). Between STH and CZY, 63% of the whiteflies selected STH; just 37% were attracted to CZY (χ^2^ = 6.40, *P* = 0.011). Similar results were observed in the wind tunnel experiments, though the response percentages were slightly lower compared with the olfactometer bioassays (**Figure 1B**). Whiteflies exhibited a significant attraction to YG over STH (χ^2^ = 17.38, *P* < 0.001) and CZY (χ^2^ = 62.40, *P* < 0.001).

### Two-Choice Assay for Intact Eggplant

**FIGURE 2:**
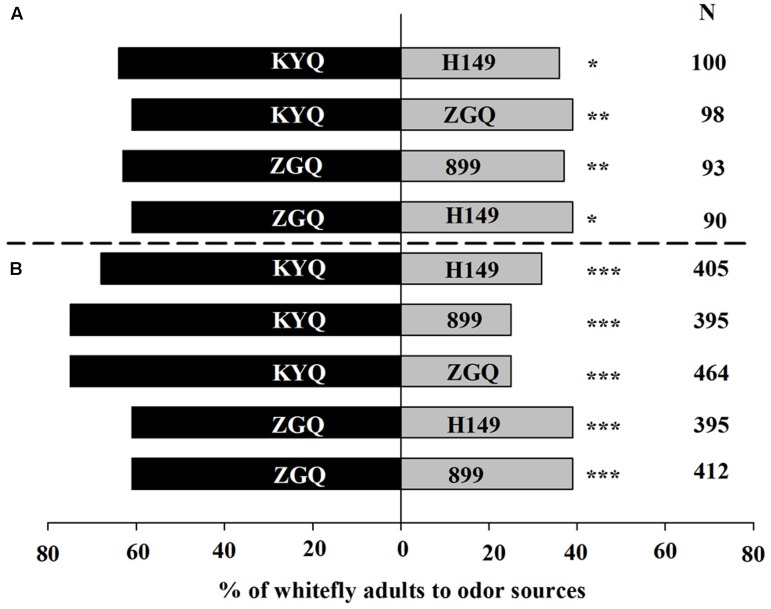
Preference of *Trialeurodes vaporariorum* to four different eggplant cultivars in **(A)** Y-tube olfactometer and **(B)** wind tunnel. Odor sources are intact *Solanum melongena* cultivar H149, 899-1-2-2-2 (899), Zi Guan Qie (ZGQ), and Kuai Yuan Qie (KYQ). The numbers to the right of the bars (N) give the number of responding individuals. The asterisks indicate a significant preference for one of the two odor sources (χ^2^ test: ^∗^*P* < 0.05, ^∗∗^*P* < 0.01, ^∗∗∗^*P* < 0.001).

Overall, 95% of the whiteflies responded to eggplant in the Y-tube; in the wind tunnel, 80% of the whiteflies made a choice. Among the four eggplant cultivars, KYQ was the most attractive, followed by ZGQ, 899, and H149 in both Y-tube and wind tunnel experiments. In the Y-tube experiments, 64% of the whiteflies were attracted toward KYQ, compared with 36% toward ZGQ (χ^2^ = 8.00, *P* = 0.005) (**Figure 2A**). In the wind tunnel, 75% of the whiteflies selected KYQ and only 25% of the whiteflies were observed on ZGQ plants (χ^2^ = 116.00, *P* < 0.001) (**Figure 2B**).

### Two-Choice Assay for Intact versus Infested Plants

**FIGURE 3:**
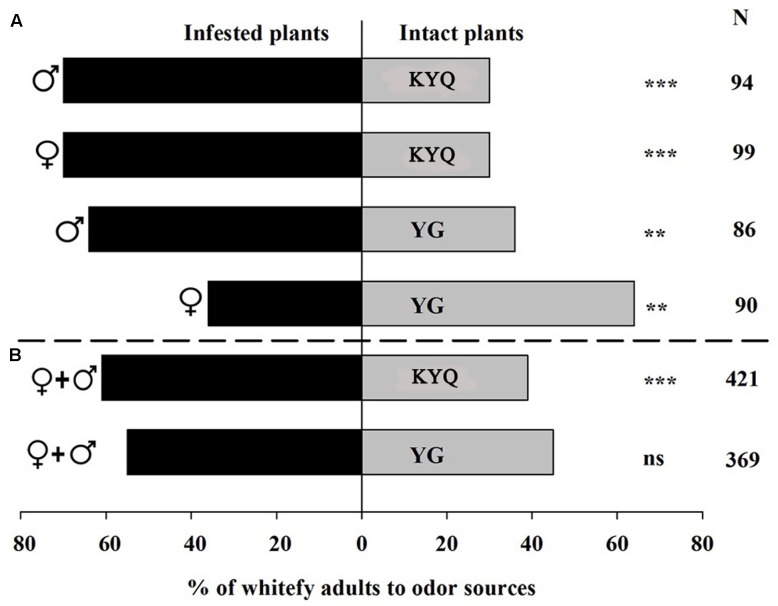
Preference of *Trialeurodes vaporariorum* in **(A)** Y-tube olfactometer and **(B)** wind tunnel. Odor sources are infested versus intact *Solanum lycopersicum* cultivar YG and *Solanum melongena* cultivar KYQ. The numbers to the right of the bars (N) give the number of responding individuals. The asterisks indicate a significant preference for one of the two odor sources (χ^2^ test: ^∗∗^*P* < 0.01, ^∗∗∗^*P* < 0.001 and ns = not significant difference). 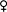 = female whiteflies, 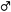 = male whiteflies, 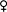 + 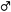 = both female and male whiteflies.

Whitefly males showed a distinct (64%) preference for the odor blends released from infested YG plants over intact YG plants (χ^2^ = 6.70, *P* = 0.010) in the Y-tube experiments. However, 64% of the female whiteflies were attracted to intact plants (χ^2^ = 7.51, *P* = 0.006), a pattern completely different from the males (**Figure 3A**). In the wind tunnel experiments, male and female whiteflies were released at the same time, and no significant preference was observed toward infested YG in the wind tunnel (**Figure 3B**). In the Y-tube experiments for eggplants, 70% of males (χ^2^ = 15.36, *P* < 0.001) and 70% of females (χ^2^ = 15.36, *P* < 0.001) chose infested KYQ over intact KYQ (**Figure 3A**). Consequently, in the wind tunnel experiments, 61% (χ^2^ = 20.54, *P* < 0.001) of whiteflies selected infested KYQ over intact plants (**Figure 3B**).

### Two-Choice Assay between the Most Attractive Tomato and Eggplant Cultivars

**FIGURE 4:**
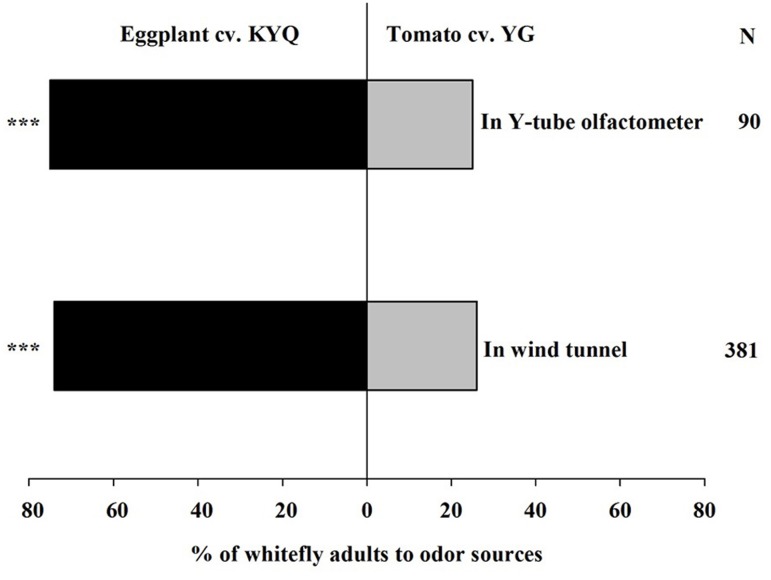
Preference of *Trialeurodes vaporariorum* in Y-tube olfactometer and the wind tunnel. Odor sources are intact *Solanum melongena* cultivar KYQ versus *Solanum lycopersicum* cultivar YG. The numbers to the right of the bars (N) give the number of responding individuals. The asterisks indicate a significant preference for one of the two odor sources (χ^2^ test: ^∗∗∗^*P* < 0.001).

The most whitefly attractive eggplant, KYQ, and tomato, YG, were selected to further examine the preferences of whiteflies. In the Y-tube, 75% (χ^2^ = 23.511, *P* < 0.001) of whiteflies, and 74% (χ^2^ = 87.90, *P* < 0.001) of whiteflies in the wind tunnel, showed a remarkable preference for KYQ over YG (**Figure 4**).

### Conspecific Lure Experiment in Y-tube Olfactometer

**FIGURE 5:**
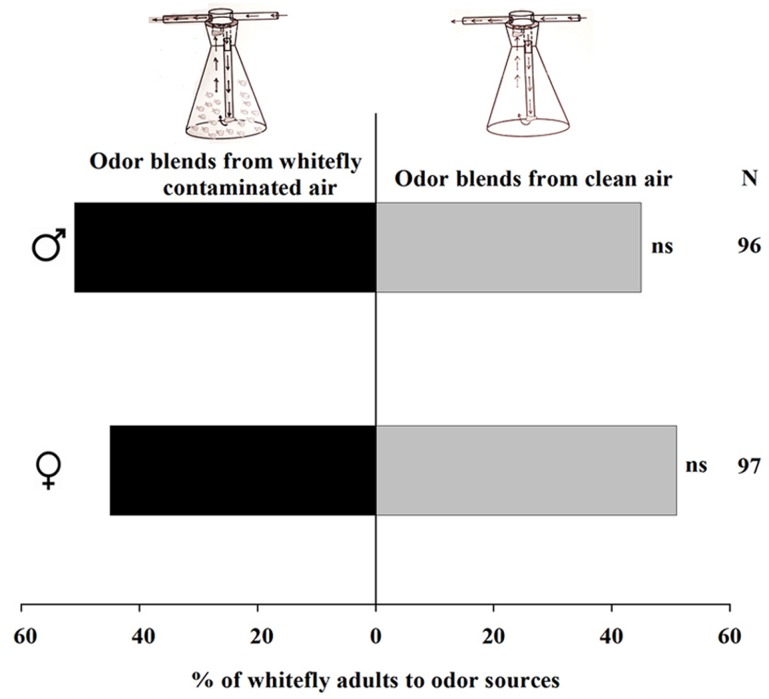
Preference of *Trialeurodes vaporariorum* in Y-tube olfactometer. Odor sources are whitefly contaminated versus clean air. The numbers to the right of the bars (N) give the number of responding individuals. (χ^2^ test: ns, not significant difference). 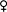 = female whiteflies, 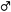 = male whiteflies.

We examined the ability of whiteflies to attract their conspecifics because olfactometer experiments were carried out for infested plants without removing whiteflies from the plants. When air that was odor-loaded by 200 whiteflies from mixed sexes was offered versus clean air to male and female whiteflies separately, neither males (χ^2^ = 0.37, *P* = 0.54) nor females (χ^2^ = 0.37, *P* = 0.54) showed any significant attraction to the odor-loaded air over the clean air (**Figure 5**).

### Free-Choice Assay

**FIGURE 6:**
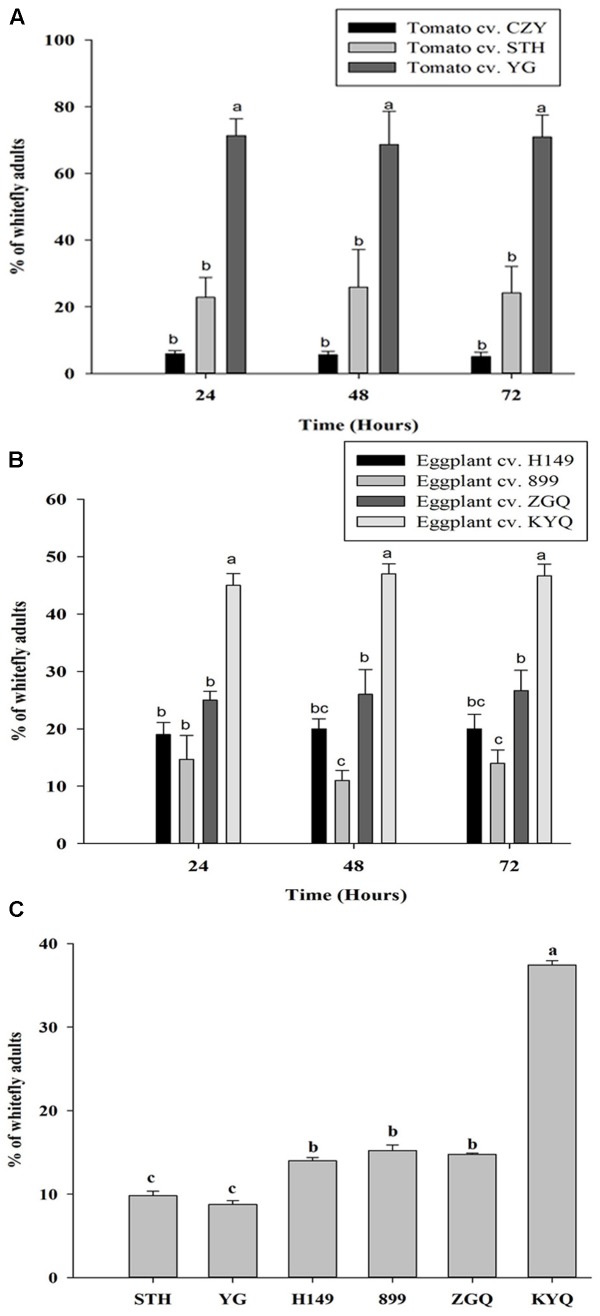
Preference of *Trialeurodes vaporariorum* in free-choice assays in net cages to **(A)** different tomato cultivars, **(B)** different eggplant cultivars within 24, 48, and 72 h and **(C)** multi-choice assays in greenhouse to *Solanum lycopersicum* cultivar STH and YG and *Solanum melongena* cultivar H149, 899, ZGQ, and KYQ. Bars represent the mean number (+SE) of whitefly adults on each plant and bars with different letters showed the statistical difference.

In this bioassay, preferences of whiteflies to intact tomato and eggplant cultivars were examined over a 72 h after a release of them in the net cage. The preference of whiteflies did not change with time, but they showed significant initial attraction to specific cultivars within a plant species during the first 24 h; they were remarkably attracted to YG among the tomato cultivars (*F* = 118.019; df = 2, 51; *P* < 0.001) and KYQ among the eggplant cultivars (*F* = 108.015; df = 3, 32; *P* < 0.001) (**Figures 6A,B**).

### Multi-Choice Assay

When we offered the two tomato cultivars STH and YG and the four eggplant cultivars H149, 899, ZGQ, and KYQ to whiteflies in the greenhouse, they preferred KYQ over the other five cultivars. The two tomato cultivars were less preferred with comparison to the three eggplant cultivars H149, 899, and ZGQ (*F* = 76.854, df = 5, 12, *P* < 0.001) (**Figure 6C**).

### Headspace Volatile Analysis of Intact Plants

**FIGURE 7:**
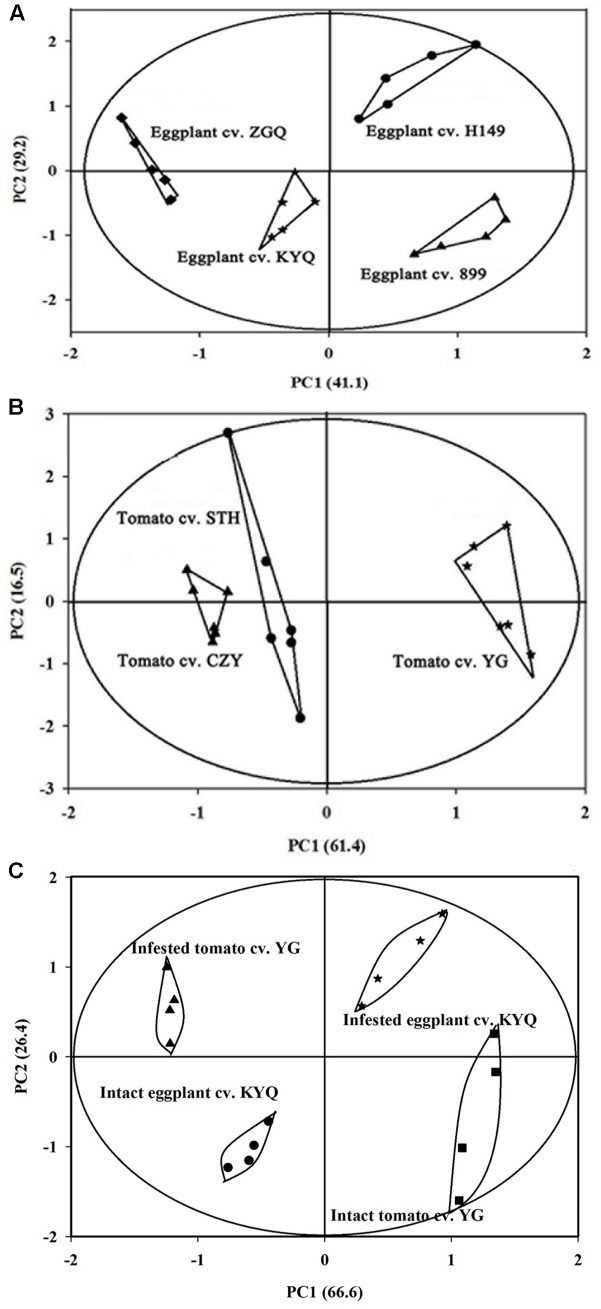
Principal component analysis (PCA) for **(A)** four *Solanum melongena* cultivar H149 (circles), 899 (triangles), ZGQ (diamonds) and KYQ (stars) based on the 21 different chemical compounds, **(B)** three *Solanum lycopersicum* cultivar CZY (triangles), STH (circles), and YG (stars) based on the 26 different chemical compounds and **(C)** infested and intact plants of tomato cultivar YG and eggplant cultivar KYQ based on the seven volatile compounds (measured as relative abundances of total peak area in individual cultivar in SPME headspace volatile collection).

The volatiles produced by eggplant and tomato plants were mainly GLVs and terpenes. Twenty-six major volatile compounds were detected in tomato plants and 21 were detected in eggplants; in both species, there were some volatile compounds that could not be detected in all cultivars (Supplementary Tables 1, 2). A PCA analysis for volatiles emitted by tomato cultivars showed a clear separation at the cultivar level, with the first principal component, separating YG from the other two cultivars, explaining 61.4% of the variance. The second component (explaining 16.5%) did not separate CZY from STH (**Figure 7B**). Similarly, the first component for eggplant cultivars separated H149 and 899 from KYQ and ZGQ and explained 41.1% of the variance, and the second component (which explained 29.2%) separated KYQ from ZGQ and also H149 from 899 (**Figure 7A**).

Seven major volatile constituents were selected for evaluating on the basis of availability of tomato and eggplants and also infested versus intact YG and KYQ. Among the volatile constituents of different tomato cultivars, the most attractive cultivar, YG, had remarkably high amounts of Z-3-hexen-1-ol, α-humulene, (E)-β-caryophyllene, and 1,1-dimethyl-3-methylene-2-vinylcyclohexane compared with other cultivars. However, the amounts of α-pinene and azulene were significantly low in YG (**Table 1**). The chemical compounds Z-3-hexen-1-ol, α-humulene, (E)-β-caryophyllene were higher in eggplant cultivar KYQ than in other cultivars. In contrast, α-pinene, azulene and 1,1-dimethyl-3-methylene-2- vinylcyclohexane were comparatively lower in KYQ (**Table 2**).

**Table 1:** Volatile chemical composition of different tomato plant cultivars.

Volatiles	Relative proportions of volatiles		
	CZY	STH	YG
Z-3-Hexen-1-ol	N.D.	3.75 ± 0.44b	12.92 ± 0.44a
a-Pinene	4.07 ± 0.27b	9.81 ± 0.96a	2.79 ± 0.75b
a-Humulene	1.95 ± 0.14b	1.35 ± 0.19b	13.37 ± 2.79a
(E)-β-Caryophyllene	0.21 ± 0.05b	0.29 ± 0.08b	3.86 ± 0.14a
Methoxyphenyl oxime	5.35 ± 0.18	5.47 ± 0.35	5.85 ± 0.50
Azulene	5.37 ± 0.80ab	6.97 ± 0.60a	4.33 ± 0.25b
1,1-Dimethyl-3-methylene-2-vinylcyclohexane	2.23 ± 1.50b	2.86 ± 1.34b	11.58 ± 2.13a

*Values are Mean + SE (six replicates). Means followed by the same letters within each row are not significantly different at *P* = 0.05 level (LSD). N.D. means compound not detected*.

**Table 2:** Volatile chemical composition of different eggplant cultivars.

Volatiles	Relative proportions of volatiles			
	H149	899	ZGQ	KYQ
Z-3-Hexen-1-ol	5.50 ± 0.68b	3.14 ± 0.79b	3.48 ± 0.54b	9.11 ± 1.03a
α-Pinene	2.91 ± 0.42c	8.39 ± 0.83a	3.49 ± 0.46bc	5.62 ± 0.46b
α-Humulene	1.76 ± 0.40b	N.D.	2.67 ± 0.68b	13.37 ± 1.23a
(E)-β-Caryophyllene	N.D.	N.D.	10.41 ± 1.32a	9.59 ± 1.47a
Methoxyphenyl oxime	5.99 ± 0.58a	5.52 ± 0.36ab	3.51 ± 0.76b	4.98 ± 0.65ab
Azulene	5.59 ± 1.11ab	7.94 ± 0.72a	2.01 ± 0.28c	4.46 ± 0.81bc
1,1-Dimethyl-3-methylene-2-	9.77 ± 0.83a	8.81 ± 0.95a	0.74 ± 0.32b	1.07 ± 0.46b
vinylcyclohexane				

*Values are Mean + SE (five replicates). Means followed by the same letters within each row are not significantly different at *P* = 0.05 level (LSD). N.D. means compounds not detected*.

### Volatiles of Infested Plants

**FIGURE 8:**
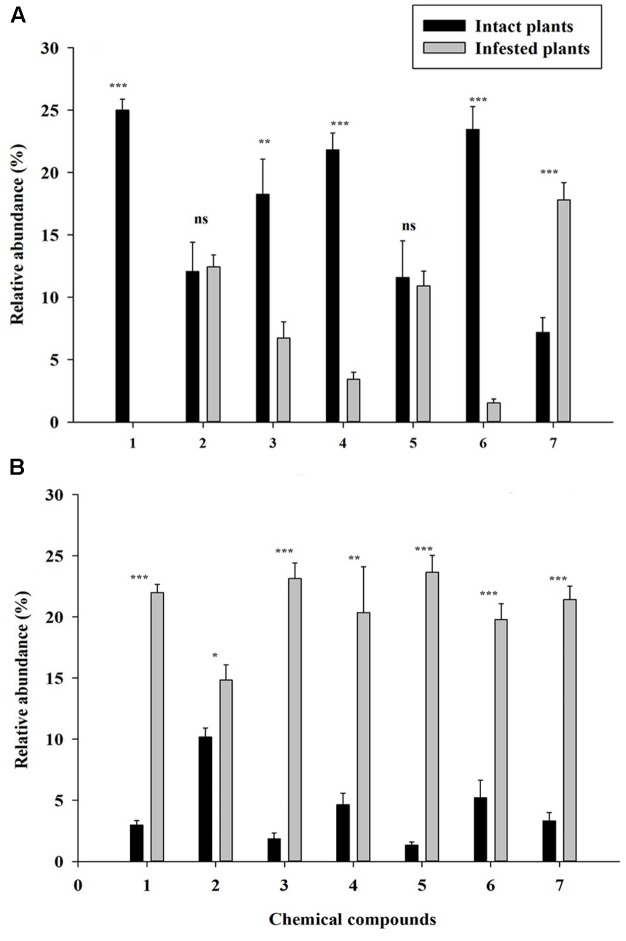
Quantitative difference of the identified volatile compounds emitted by intact (black bars) and infested (gray bars) plants. Each bar shows the relative amounts of (±SE) collected headspace from seven plants of 24 h whitefly infested and intact plants of **(A)** tomato cultivar YG and **(B)** eggplant cultivar KYQ. (1) Z-3-hexen-1-ol, (2) a-pinene, (3) a-humulene, (4) (E)-β-caryophyllene, (5) methoxyphenyl oxime, (6) azulene, and (7) 1,1-dimethyl-3-methylene-2-vinylcyclohexane. The asterisks indicate a significant difference of the volatile compound between intact and infested plants (^∗^*P* < 0.05, ^∗∗^*P* < 0.01, ^∗∗∗^*P* < 0.001, ns, not significant difference).

The PCA analysis showed a clear separation of volatile productions of infested plants of both YG and KYQ compared with intact plants of the same cultivars as well as at the different cultivar levels (**Figure 7C**). The emitted quantities of Z-3-hexen-1-ol, α-humulene, E-β-caryophyllene and azulene declined in infested YG compared to intact plants while 1,1-dimethyl-3-methylene-2-vinylcyclohexane emission was increased by the whitefly infestation. However, α-pinene and methoxyphenyl oxime did not show any quantitative difference after whitefly infestation (**Figure 8A**). In contrast, eggplant cultivar KYQ showed a remarkable increase in volatile emissions of Z-3-hexen-1-ol, α-pinene, α-humulene, (E)-β-caryophyllene, methoxyphenyl oxime, azulene and 1,1-dimethyl-3-methylene-2-vinylcyclohexane by infested plants (**Figure 8B**).

## Discussion

Evaluation of the behavioral response of *T. vaporariorum* for olfactory and visual stimuli emitted from plants might be the best strategy to develop whitefly management programs using attractive chemical constituents. Although eggplants have been used as a trap crop for controlling *T. vaporariorum* (Lee et al., 2010; Moreau and Isman, 2010), the mechanisms of host plant selection of *T. vaporariorum* based on the surrounding volatile cues are not well understood yet (Tosh and Brogan, 2015). However, the whiteflies’ responses toward plant visual cues are well-studied and have been suggested as an important factor in the selection of host plants (Avery et al., 2015). The presence of both visual and olfactory cues may have a synergistic effect on herbivore decision-making in finding a suitable host (Domingue et al., 2016).

We found that *T. vaporariorum* had a significant preference for intact YG over other tomato cultivars, whereas KYQ attracted the largest quantity of whiteflies among the tested eggplant cultivars. Subsequently, we demonstrated a greater attractiveness of whiteflies to KYQ over YG in both Y-tube olfactometer and wind tunnel experiments. Fascinatingly, when we offered all plants in free-choice and multi-choice assays, the whiteflies’ initial decision-making ability was consistent with this, as they quickly chose the most-attractive plants as determined by both Y-tube and wind tunnel bioassays. When initially targeting a host, the sweet potato whitefly *Bemisia tabaci* (Gennadius) (Hemiptera: Aleyrodidae) primarily considers plant olfactory cues (Bleeker et al., 2009). Therefore, a potential explanation for our results is that *T. vaporariorum* may also use olfaction as the primary stimulus at the initial host plant targeting, and that the quantity of volatile emissions from KYQ is strong enough to enhance its attractiveness to *T. vaporariorum* over other plants.

Phloem-feeding insects are responsible for regulating the salicylic acid (SA) pathway (Zhang et al., 2009; Cao et al., 2016). *T. vaporariorum* is a phloem-feeding insect, and therefore may interfere with the SA signaling pathway of tested plant cultivars. Some studies have pointed out that plants infested by the whitefly *B. tabaci* attract large quantities of heterospecific herbivores (Zhang et al., 2009). It is likely that *T. vaporariorum* also have the ability to change volatile emissions by infested plants from those by undamaged plants. *T. vaporariorum*-infested bean plants emit quantitatively different volatile blends than intact plants, though this has been investigated only for indirect defense (Birkett et al., 2003). We observed that infested KYQ induced the attraction of more whiteflies, suggesting that even when damage is caused by the same insect species, plant species and cultivars play an important role in determining the attractiveness to whiteflies. Therefore, the response of *T. vaporariorum* to the VOCs induced by their conspecific-infested plants can be considered innate. Nevertheless, similar observations have been reported for conspecific attraction by the Colorado potato beetle (*Leptinotarsa decemlineata* Say) (Coleoptera: Chrysomelidae) on potato plants (Bolter et al., 1997) and spider mites (*Tetranychus urticae* Koch) (Trombidiformes: Tetranychidae) on lima bean plants (Horiuchi et al., 2003). Our study provides evidence that conspecific-induced volatiles could attract *T. vaporariorum*.

Finding a suitable oviposition site for offspring, including avoidance of conspecific-infested plants, is a major task of mothers for preventing competition (De Moraes et al., 2001; Saad et al., 2013). Some female herbivores use defensive mimicry by the plants to eliminate conspecific-infested plants from consideration and thus to protect themselves from exposure to natural enemies (Nomikou et al., 2003). In our experiments, tomato cultivar YG repelled the females after conspecific infestation, which may be in line with these studies. However, it is surprising that conspecific-induced volatiles from infested eggplant KYQ attracted both males and females; this aggregation behavior was shown for the tea weevil (*Myllocerinus aurolineatus* Voss) (Coleoptera: Curculionidae), which preferred conspecific-infested tea plants (Sun et al., 2010). Further, females of the spider mite *Tetranychus evansi* (Baker and Pritchard) (Acarina: Tetranychidae) preferred to select conspecific-infested tomato plants, considering them an appropriate host for offspring development (Sarmento et al., 2011). It seems that individual plant species produce specific blends of chemical compounds after infestation that may either repel or attract not only females but males as well. Therefore, we can suggest that female whiteflies strongly consider the quality of plants as both food sources and enemy-free areas for their offspring, and males search for putative mating partners by attracting them to infested plants, as some conspecific-infested plants produce volatile cues for female aggregation, and meanwhile emit kairomones that attract males as well (Frati et al., 2009). Our Y-tube bioassays confirmed that whiteflies did not have any remarkable ability to attract their conspecifics, and this phenomenon also reported for tea weevils even though they aggregate on conspecific-infested plants (Sun et al., 2010). The VOCs may, therefore, be plant-derived volatiles resulting from whitefly infestation, not from the whiteflies themselves to attract conspecifics.

Different cultivars of the same plant species emit different plant volatiles (Gols et al., 2008; Bleeker et al., 2009; Poelman et al., 2009). Hence, one of the paramount questions to be addressed here was whether different plant cultivars within individual species are categorically different in their emitted volatiles. The PCA analysis demonstrated a statistical separation of different plant cultivars of both tomato and eggplant based on the headspace volatile compounds. Moreover, the majority of headspace volatile compounds of intact tomato and eggplant plants were different, with a few exceptions such as (Z)-3-hexen-1-ol, α-pinene, (E)-β-caryophyllene, α-humulene, azulene, methoxyphenyl oxime, and 1,1-dimethyl-3-methylene-2-vinylcyclohexane, as different plant species produce different volatile blends (Takabayashi and Dicke, 1996). However, terpenoids [α-pinene, (E)-β-caryophyllene, α-humulene, azulene] and GLVs [(Z)-3-hexen-1-ol] were the most abundant volatiles and considered as important chemicals due to their constitutive relationship between the most attractive eggplant and tomato cultivars and also infested versus intact plants of these cultivars. The active components of plant volatiles are complicated, and simply selecting major compounds will not be enough to change the behavior of whiteflies, since herbivore host selection behavior is a complex process involving different blends of semiochemicals and minor compounds can also have divers effect on insect attraction or repellent properties (Dicke, 2000; Clavijo et al., 2014).

Our results demonstrated that plant infestation by *T. vaporariorum* played a key role in the changes of plant volatile emissions since PCA analysis demonstrated the clear separation of infested plants from intact plants. Additionally, the headspace volatile analysis proved quantitative differences in (Z)-3-hexen-1-ol, α-humulene, (E)-β-caryophyllene and azulene emissions of whitefly infested YG and KYQ plants compared with their intact plants. Some plants emit diverse mixtures of volatiles depending upon the type of damage (Rojas, 1999; Degenhardt et al., 2003) and the plant species (Pare and Tumlinson, 1999). Our results were consistent with (Z)-3-hexen-1-ol emission after infestation by *T. vaporariorum* on bean plants (Birkett et al., 2003), (E)-β-caryophyllene from *Lygus rugulipennis* (Poppius) (Hemiptera: Miridae) damaged *Vicia faba* plants (Frati et al., 2009), and α-humulene production of leaf beetle *Oreina cacaliae* Schrank (Coleoptera: Chrysomelidae)-infested *Petasites paradoxus* (Kalberer et al., 2001), which led to conspecific attraction. (Z)-3-hexen-1-ol directly manipulates the behavioral response of different herbivores (Wei and Kang, 2011; Domingue et al., 2016), and interestingly, (Z)-3-hexen-1-ol could not be found in the infested YG whereas this C6-green leaf volatile compound was remarkably increased in infested KYQ. The same results were observed for (E)-β-caryophyllene, α-humulene, and azulene terpenes in infested versus intact YG and KYQ as terpenoids play a major role in *B. tabaci*–tomato plant interaction (Bleeker et al., 2009). Although infested KYQ showed remarkable ability to produce α-pinene over intact plants for attracting both male and female *T. vaporariorum*, it is early to confirm that whether it has a synergistic effect on whiteflies’ host plant selection. However, we can assume that this compound play a significant role in the plants’ attractive or repellent properties of both male and female whiteflies, such as plant derived α-pinene has a specific ability to attract both sexes of Olive fruit fly (*Bactrocera oleae* Rossi) (Diptera: Tephritidae) (Gerofotis et al., 2013). We cannot simply explain the relationship of these volatile constituents to the behavioral response of *T. vaporariorum* since (a) it is a polyphageous species (Rezaei et al., 2015) which has a preference for a large number of volatile blends, (b) their natural ecosystems consist of different mixtures of VOCs, and therefore we must select a suitable volatile constituent to include in a sound management package, and (c) the selected volatile must have a high capacity for changing the behavior of the herbivore at the olfactory level with a minimal dosage (Corrado et al., 2007).

In summary, our experiments obtained highly impressive results showing that KYQ is the most attractive plant cultivar of those tested and that infested KYQ could attract a significantly larger quantity of both male and female whiteflies than could intact plants. Therefore, more experiments must be performed for further clarifications to improve KYQ as a trap crop. Our study demonstrated that (Z)-3-hexen-1-ol, α-pinene, (E)-β-caryophyllene, α-humulene, and azulene as important cues for behavioral changes of *T. vaporariorum*. Even though we have confirmed that significant numbers of whiteflies were attracted to these plants based on the major five volatile compounds, the volatile blends responsible for attracting such a large quantity of whiteflies in odor-mixed natural conditions are still difficult to determine. Hence, to utilization of selected volatiles for developing sustainable semiochemical management, further experiments should be performed to get confirm the volatile compound composition that significantly attracts whiteflies under greenhouse and field conditions.

## Author Contributions

Conceived and designed the experiments: HD and T-XL. Performed the experiments: HD, HR, NA, Z-FZ, Y-HL. Analyzed the data: HD. Wrote the paper: HD and T-XL.

## Conflict of Interest Statement

The authors declare that the research was conducted in the absence of any commercial or financial relationships that could be construed as a potential conflict of interest.

## Supplementary Material

FIGURE S1 Five weeks old eggplant cv. KYQ in 3 L glass jar for Y-tube olfactometer set-up arrangement.
