# Supplementary figures and images for: Volatile-Mediated Attraction of Greenhouse Whitefly Trialeurodes vaporariorum to Tomato and Eggplant
Source: Front Plant Sci. 2017 Jul 20;8:1285. doi: 10.3389/fpls.2017.01285 (PMC5517405; doi:10.3389/fpls.2017.01285)

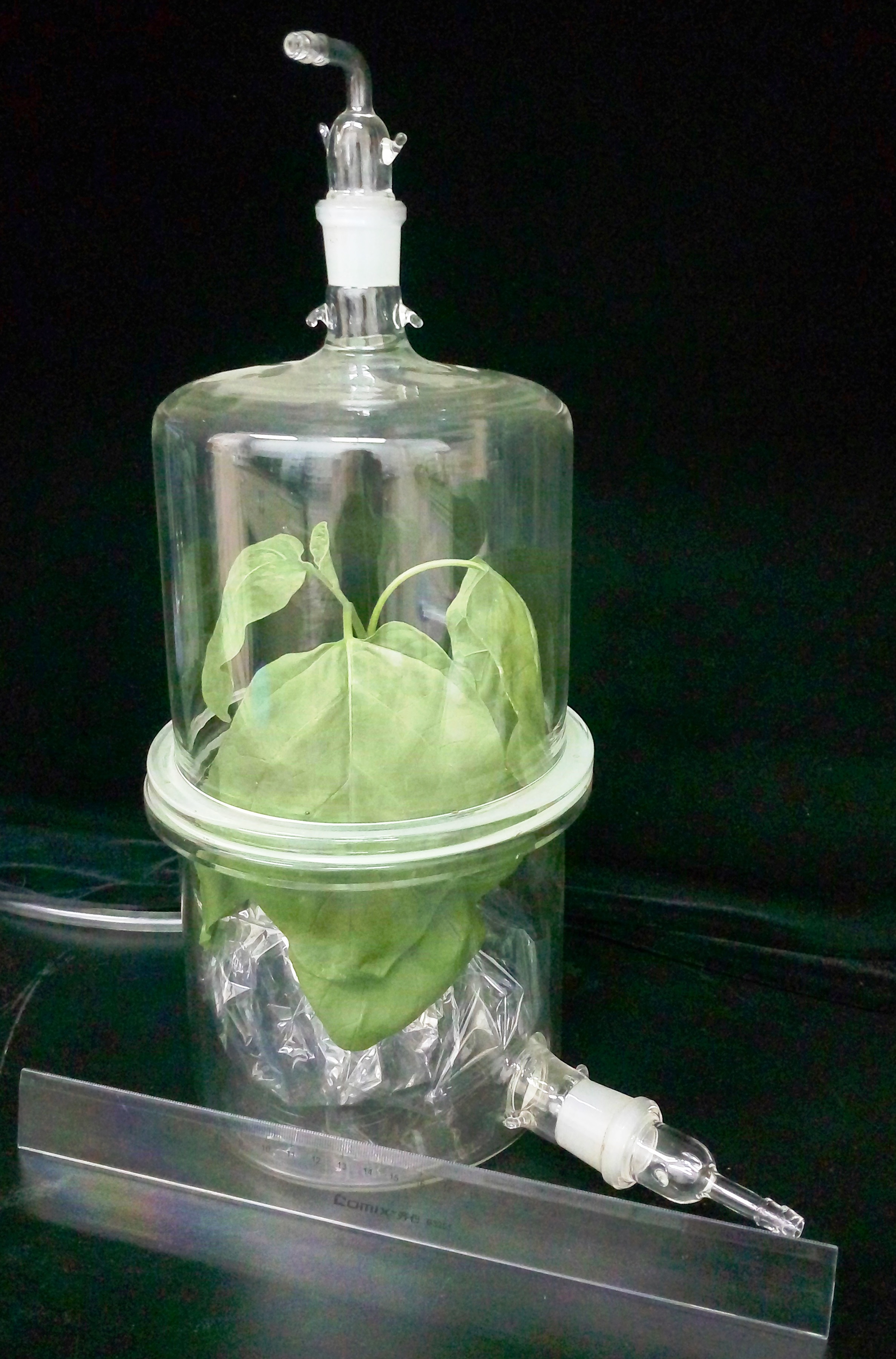

Supplement: FIGURE S1 — Five weeks old eggplant cv. KYQ in 3 L glass jar for Y-tube olfactometer set-up arrangement. [file Image_1.JPEG]
