# Supplementary material for: Volatile-Mediated Attraction of Greenhouse Whitefly Trialeurodes vaporariorum to Tomato and Eggplant
Source: Front Plant Sci. 2017 Jul 20;8:1285. doi: 10.3389/fpls.2017.01285 (PMC5517405; doi:10.3389/fpls.2017.01285)
Supplement: Supplementary file 2 [file Table_1.DOCX]

**Supplementary Table 1: Volatile chemical composition of different tomato cultivars**

**Volatiles Relative proportions of volatiles CZY STH YG**

| Z-3-Hexen-1-ol | N.D. | 3.75±0.44**b** | 12.92±0.44**a** | |
| --- | --- | --- | --- | --- |
| 2-Thujene | 0.84±0.11**b** | 1.15±0.20**b** | 14.67±1.06**a** | |
| α−Pinene | 4.07±0.27**b** | 9.81±0.96**a** | 2.79±0.75**b** | |
| β-Pinene | 4.67±0.48**b** | 11.77±0.87**a** | 0.22±0.07**c** | |
| (+)-3-carene | 3.35±0.25**b** | 9.84±0.68**a** | 3.48±0.47**b** | |
| (+)-4-carene | 0.16±0.03**b** | 0.28±0.03**b** | 16.22±4.00**a** | |
| C-Terpinene | 4.01±0.90**b** | 9.93±1.93**a** | 2.73±0.23**b** | |
| O-Cymene | 0.95±0.06**b** | 3.68±0.23**b** | 12.04±1.72**a** | |
| β-Ocimene | N.D. | 0.01±0.01**b** | 16.83±1.94**a** | |
| (E)-β-caryophyllene | 0.21±0.05**b** | 0.29±0.08**b** | 3.86±0.14**a** | |
| Caryophyllene Oxide | N.D. | 1.24±0.21**b** | 16.43±1.22**a** | |
| α−Humulene | 1.95±0.14**b** | 1.35±0.19**b** | 13.37±2.79**a** | |
| α-Copaene | 3.00±0.86 | 6.28±1.80 | 4.99±0.71 | |
| δ-elemene | N.D. | N.D. | 16.00±0.80**a** | |
| Azulene | 5.37±0.80**ab** | 6.97±0.60**a** | 4.33±0.25**b** | |
| Phellendrene | 0.38±0.08**b** | 1.67±0.07**b** | 14.61±1.73**a** | |
| α-Myrcene | 0.14±0.02**b** | 0.42±0.09**b** | 16.11±2.81**a** | |
| Camphene | 7.72±1.23**a** | 9.29±0.89**a** | N.D. | |
| Limonene | 2.62±0.54**b** | 4.50±0.81**b** | 9.54±0.69**a** | |
| Trans-sesquisabinene | N.D. | 4.35±1.06**b** | 12.32±1.03**a** | |
| Methyl Salicilate | N.D. | 0.52±0.12**b** | 16.15±0.78**a** | |
| Decanal | 8.75±1.10**a** | 1.50±0.40**b** | 6.43±0.82**a** | |
| Undecane | 8.62±0.85**a** | 3.39±0.96**b** | 4.66±0.50**b** | |
| Dodecane | 5.95±0.53 | 5.99±1.20 | 4.56±0.13 | |
| Methoxyphenyl Oxime | 5.35±0.18 | 5.47±0.35 | 5.85±0.50 | |
| 1,1-Dimethyl-3-Methylene-2- | 2.23±1.50**b** | 2.86±1.34**b** | 11.58±2.13**a** | |
| vinylcyclohexane |  |  | |  |

Values are Mean+SE (Six replicates). Means followed by the same letters within each row are not significantly different at P=0.05 level (LSD). N.D. means compound not detected.
