# Supplementary material for: Volatile-Mediated Attraction of Greenhouse Whitefly Trialeurodes vaporariorum to Tomato and Eggplant
Source: Front Plant Sci. 2017 Jul 20;8:1285. doi: 10.3389/fpls.2017.01285 (PMC5517405; doi:10.3389/fpls.2017.01285)
Supplement: Supplementary file 3 [file Table_2.DOCX]

**Supplementary Table 2: Volatile chemical composition of different eggplant cultivars**

**Volatiles Relative proportions of volatiles**

**H149 899 ZGQ KYQ**

| 1-Hexanol | | 7.68±0.84**a** | | 8.52±1.05**a** | 0.07±0.04**c** | | 3.73±0.58**b** | |  |
| --- | --- | --- | --- | --- | --- | --- | --- | --- | --- |
| 3-hexen-1-ol | | 18.27±1.64**a** | | 1.63±0.41**b** | 0.19±0.08**b** | | N.D. | |  |
| Z-3-hexen-1-ol | | 5.50±0.68**b** | | 3.14±0.79**b** | 3.48±0.54**b** | | 9.11±1.03**a** | |  |
| Z-3-Hexenyl Acetate | | 7.75±1.11**a** | | 3.12±0.74**b** | 2.61±0.37**b** | | 5.93±1.26**ab** | |  |
| Z-2-Hexanyl Acetate | | 3.45±0.66**b** | | 1.02±0.30**b** | 3.03±0.73**b** | | 12.50±0.89**a** | |  |
| α-Pinene | | 2.91±0.42**c** | | 8.39±0.83**a** | 3.49±0.46**bc** | | 5.62±0.46**b** | |  |
| α-Ocimene | | 4.13±0.99 | | 5.36±1.30 | 5.16±0.99 | | 5.36±1.43 | |  |
| Trans-α-Bergamotene | | 3.56±0.95**b** | | 1.73±0.30**b** | 3.23±0.79**b** | | 11.48±1.16**a** | |  |
| β-Linalool | | 4.99±0.57**ab** | | 7.22±0.86**a** | 4.14±0.73**b** | | 3.64±0.73**b** | |  |
| α- Humulene | | 1.76±0.40**b** | | N.D. | 2.67±0.68**b** | | 13.37±1.23**a** | |  |
| α-Farnesene | | 1.03±0.66**b** | | 12.50±1.19**a** | 3.10±0.79**b** | | 3.15±0.46**b** | |  |
| (E)-β-caryophyllene | | N.D. | | N.D. | 10.41±1.32**a** | | 9.59±1.47**a** | |  |
| Dimethyl Phathalate | | 6.06±0.97**ab** | | 8.73±0.98**a** | N.D. | | 5.21±0.96**b** | |  |
| Indole | | 6.63±1.07**b** | | 12.42±1.22**a** | 1.00±0.21**c** | | N.D. | |  |
| Decanal | | 6.94±0.53**a** | | 3.84±0.65**b** | 1.15±0.29**c** | | 7.27±0.62**a** | |  |
| Dodecane | | 3.67±0.63 | | 5.53±1.44 | 4.16±0.56 | | 6.19±0.70 | |  |
| Z-2-Methylbutanal oxime | | 8.33±0.80**a** | | 3.17±0.80**b** | 5.77±1.05**ab** | | 2.72±0.33**b** | |  |
| Methoxyphenyl oxime | | 5.99±0.58**a** | | 5.52±0.36**ab** | 3.51±0.76**b** | | 4.98±0.65**ab** | |  |
| Azulene | | 5.59±1.11**ab** | | 7.94±0.72**a** | 2.01±0.28**c** | | 4.46±0.81**bc** | |  |
| Camphor | | 1.54±0.37**b** | | 13.25±1.03**a** | 2.74±0.37**b** | | 2.47±0.29**b** | |  |
| 1,1-Dimethyl-3-Methylene-2- | | 9.77±0.83**a** | | 8.81±0.95**a** | 0.74±0.32**b** | | 1.07±0.46**b** | |  |
| vinylcyclohexane |  | |  | | |  | |  | |

Values are Mean+SE (Five replicates). Means followed by the same letters within each row are not significantly different at P=0.05 level (LSD). N.D. means compounds not detected.
